# Determination of collective behavior of the financial market

**DOI:** 10.1186/s40064-016-3203-4

**Published:** 2016-09-13

**Authors:** Shouwei Li, Tao Xu, Jianmin He

**Affiliations:** School of Economics and Management, Southeast University, Nanjing, 211189 China

## Abstract

In this paper, we adopt the network synchronization to measure the collective behavior in the financial market, and then analyze the factors that affect the collective behavior. Based on the data from the Chinese financial market, we find that the clustering coefficient, the average shortest path length and the volatility fluctuation have a positive effect on the collective behavior respectively, while the average return has a negative effect on it; the effect of the average shortest path length on the collective behavior is the greatest in the above four variables; the above results are robust against the window size and the time interval between adjacent windows of the stock network; the effect of network structures and stock market properties on the collective behavior during the financial crisis is the same as those during other periods.

## Background

The financial market is an example of complex system, and can be well mapped as a network. For example, the connection between two stocks can be defined according to their price dynamics by taking into account the correlation between their respective time series (Peron et al. 2012). This allows analyzing the financial market based on methods and tools of the complex network theory. This methodology has been found to uncover important information about financial markets, and has shed new light on their underlying structure and dynamics (Kenett and Havlin 2015).

A lot of researchers construct networks of the financial market and analyze their statistical properties (e.g., Mantegna 1999; Vandewalle et al. 2001; Boss et al. 2004; Huang et al. 2009; Namaki et al. 2011; Markose et al. 2012; Gao et al. 2013; Wilinski et al. 2013; Nobi et al. 2015). One of the most frequently studied financial market is the stock market. For example, Mantegna (1999) observes the hierarchical structure in the US stock market based on the concept of the minimum spanning tree. Vandewalle et al. (2001) present a power-law distribution for the US stock market network during the year 1999. Gao et al. (2013) find that networks of the financial market have a small-world property. Wilinski et al. (2013) investigate structural and topological phase transitions on the German stock exchange. Nobi et al. (2015) analyze structural changes in the minimal spanning tree and the hierarchical network in the Korean stock market around the global financial crisis. Besides, there are many studies on network stability of the financial market (e.g., Peron et al. 2012; Heiberger 2014; Yan et al. 2014; Song et al. 2014; Yan et al. 2015; Heath et al. 2015; Brida et al. 2016). For instance, Peron et al. (2012) adopt an entropy-related measurement to quantify the resilience of the evolving network of the financial market, and analyze the impact of network structures on the resilience. Yan et al. (2015) investigate the topological stability of stock market network by investigating the topological robustness, namely, the ability of the network to resist structural or topological changes.

When stock prices exhibit a similar tendency, there will be the collective behavior in the financial market. There are many studies on the collective behavior in financial markets (e.g., Plerou et al. 2001; Gopikrishnan et al. 2001; Pan and Sinha 2007; Balogh et al. 2010; Maskawa 2012; Zhao et al. 2015). For example, Gopikrishnan et al. (2001) quantify and interpret the collective behavior in financial markets. Pan and Sinha (2007) analyze the collective behavior of stock price movements in an emerging market, and find that compared to developed markets, this emerging market exhibits strong correlations in the movement of stock prices. Maskawa (2012) examines the collective behavior of stock returns, and analyzes the market mode, which is a market-wide collective mode, with constituent issues of the FTSE 100 index listed on the London Stock Exchange. Zhao et al. (2015) find the financial coherence and incoherence coexistence collective behavior emerges as the system evolves into the stable state.

The collective dynamics of complex systems can be studied based on the concepts of network synchronization (Watts and Strogatz 1998). However, there are few studies on the analysis of the collective behavior in the financial market based on network synchronization. The rare instances include Peron and Rodrigues (2011) and Liu and Tse (2012). Peron and Rodrigues (2011) quantify the emergence of the collective behavior in the financial market by using concepts of the network synchronization, and analyze the effect of network structures on the collective behavior, where the analyzed network structures include the network strength heterogeneity, the clustering coefficient and the average shortest path length. Liu and Tse (2012) demonstrate synchronization in the network of stock markets, and show how it is related to market volatility.

Therefore, we know that there are many studies on the collective behavior of financial markets. However, there are few research on the collective behavior of financial markets from a network perspective. And the few studies only analyze the effect of network structures or stock market properties on the collective behavior. If we consider both network structures and stock market properties at the same time, what will be the result? Moreover, what is the effect of the latest financial crisis 2007–2008? In order to answer the above questions, in this paper, we investigate the determination of collective behavior of the financial market based on the data from the Chinese financial market. Compared with the above studies, the contributions of this paper include the investigation of the effect of both network structures and stock market properties on the collective behavior, the research on the effect of the latest financial crisis 2007–2008 on the above result, and the analysis of the robustness of the effect of both network structures and stock market properties on the window size and the time interval between adjacent windows of stock networks.

The remainder of this paper is organized as follows. After this introduction, “Methodology” section describes the methodology. “Empirical results” section presents the main results, and “Conclusion” section provides a conclusion.

## Methodology

### Measure of the collective behavior

Let $Y_{i}$ be the return of stock *i*, where $Y_{i}=lnP_{i}(t)-lnP_{i}(t-1)$ and $P_{i}(t)$ is the closing price at day *t*. We can obtain the correlation coefficient $\rho _{ij}$ between two stocks *i* and *j*, which is given as follows.

1 $$\rho _{ij}=\frac{\left\langle Y_{i}Y_{j} \right\rangle - \left\langle Y_{i} \right\rangle \left\langle Y_{j} \right\rangle }{\sqrt{\left( \left\langle Y_{i}^{2} \right\rangle - \left\langle Y_{i} \right\rangle ^{2}\right) \left( \left\langle Y_{j}^{2} \right\rangle - \left\langle Y_{j} \right\rangle ^{2}\right) }},$$

where $\langle Y \rangle$ denotes the statistical mean of *Y*. Moreover, the distance $d_{ij}$ between two stocks *i* and *j* can be calculated by the following formula.

2 $$d_{ij}=\sqrt{2\left( 1-\rho _{ij}\right) }.$$

The distances among *N* stocks form a distance matrix. Similar to the study of Peron et al. (2012), we can construct a financial market network based on this matrix, where every stock denotes a network node, and nodes *i* and *j* are connected by an edge with the weight $w_{ij}=exp(-d_{ij})$.

Let *T* be the time span for *N* stocks. Following the studies of Peron and Rodrigues (2011) and Liu and Tse (2012), we construct dynamic networks by setting a time window of length $\Delta t$ days at a $\delta$-day interval. In other words, a network is obtained by considering the time series inside each window. This window is displaced by an amount of $\delta$ days, and then a new network can be obtained. Network synchronization is used to describe the collective dynamics of complex systems (Watts and Strogatz 1998). Therefore, in this paper, we adopt network synchronization to measure the collective behavior of the financial market. For financial market networks, network synchronization means that different stocks in the financial market tend to exhibit similar behavior. In this paper, we adopt the method of Liu and Tse (2012) to analyze network synchronization, which is the average of the weights of all edges in the financial market network. Therefore, the level of the network synchronization (*S*) can be obtained from the following formula.

3 $$S=\frac{1}{N(N-1)}\sum \limits _{i=1}^{N}\sum \limits _{j=1, j\ne i}^{N}w_{ij}.$$

### Regression analysis

Peron and Rodrigues (2011) select 348 stocks from the database formed by the daily prices of 3799 stocks traded at New York Stock Exchange, and find that the clustering coefficient and the average shortest path length also contribute negatively to the collective behavior. Liu and Tse (2012) adopt the data from stock markets from 67 member countries of the World Federation of Exchanges, and find that there is a high tendency that stock markets will behave synchronously when the world stock market fluctuates. In addition, a negative correlation between average stock prices and the collective behavior is found to exist (Ramchand and Susmel 1998; Li et al. 2005; Liu and Tse 2012). Maskawa (2012) finds that the collective behavior of stock prices is correlated with the market crash. During the last decade, the Shanghai Stock Exchange exhibits higher P/E ratios and investment performance criteria than the most developed and emerging markets (Hsieh et al. 2013). It is now an important emerging market in the world, which attracts much attention (Shen and Zheng 2009; Jiang and Zheng 2012; Hsieh et al. 2013). Based on the above literature, this paper investigates how network structures and stock market properties affect the collective behavior of financial markets based on the data from the Shanghai Stock Exchange. And for this question, what is the effect of the latest financial crisis 2007–2008?

Investigating the effect of topological properties on synchronization is one of the main concerns in complex networks research (Boccaletti et al. 2006), where the clustering coefficient and the average shortest path length are studied in some works (Watts and Strogatz 1998; McGraw and Menzinger 2005; Peron and Rodrigues 2011). Therefore, in this paper topological properties investigated include the clustering coefficient (*C*) and the average shortest path length (*l*). Stock returns and their volatilities directly affect the correlation among stock price volatilities and stock networks. Therefore, similar to the study of Liu and Tse (2012), we consider stock market properties as the time-varying average return ($\mu$) and the volatility ($\sigma$).

In order to answer the above questions, we adopt the regression analysis, which is used to examine the relationship between variables. Therefore, we propose the following regression model.

4 $$S=\alpha _{0}+\alpha _{1}C+\alpha _{2}l+\alpha _{3}\mu +\alpha _{4}\Delta \sigma +\epsilon ,$$

where $\alpha$ is the unknown parameter, and $\epsilon$ is a normal distribution with mean zero and standard deviation $\varsigma$. $\Delta \sigma$ is equal to $\sigma (m)-\sigma (m-1)$, where *m* is the window sequential number. The clustering coefficient is calculated by the following formula (Onnela et al. 2005)

5 $$C=\frac{1}{N}\sum \limits _{i=1}^{N}\sum \limits _{j,k}\frac{\left( w_{ij}w_{ik}w_{jk}\right) ^{\frac{1}{3}}}{k_{i}\left( k_{i}-1\right) \max \nolimits _{p,q}w_{pq}},$$

where $k_{i}$ is the degree of stock *i* and $\max \nolimits _{p,q}w_{pq}$ is the maximum weight in the network. According to the study of Peron and Rodrigues (2011), the average shortest path length *l* is given by

6 $$l=\frac{1}{N(N-1)}\sum \limits _{i\ne j}\tau _{ij},$$

where $\tau _{ij}$ is the length of the shortest distance between stocks *i* and *j*. $\mu$ and $\sigma$ are given as follows.

7 $$\mu =\frac{1}{N}\sum \limits _{i=1}^{N}\left\langle Y_{i}\right\rangle , \sigma =\frac{1}{N}\sum \limits _{i=1}^{N}\sqrt{\left\langle Y_{i}^{2}\right\rangle -\left( \left\langle Y_{i}\right\rangle \right) ^{2}}.$$

## Empirical results

In this paper, the stock market data base corresponds to the daily closing price of the A-share market of Shanghai Stock Exchange in China. We select 279 stocks from this set, where there are historical data from 1 January 2006 to 31 December 2010 and the time of continuous suspension is no longer than 30 days. In order to analyze the effect of the financial crisis 2007–2008, the total period is subdivided into three periods: the period from January 2006 to June 2007 as the period of the normal stock market state, the period from July 2007 to December 2008 as the period of the financial crisis, and the period from January 2009 to December 2010 as the period of the stock market recovery.

**Fig. 1:**
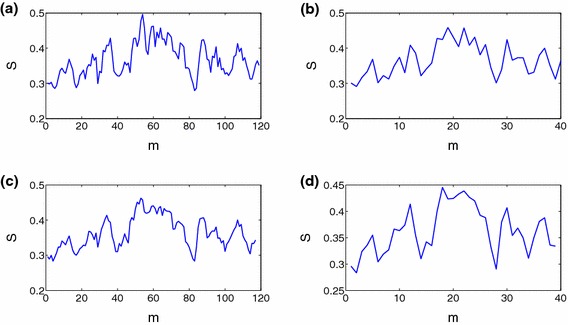
Evolution of the network synchronization about the window sequential number *m*. **a**, **b**, **c** and **d** are the results when $\Delta t=30$ and $\delta =10$, $\Delta t=30$ and $\delta =30$, $\Delta t=45$ and $\delta =10$, $\Delta t=45$ and $\delta =30$ respectively

### Collective behavior evolution

We first analyze the characteristics of the network synchronization of the financial market. Figure 1 presents the evolution of the network synchronization about the window sequential number *m*, where (a), (b), (c) and (d) are the results in the four cases, namely, $\Delta t=30$ and $\delta =10$, $\Delta t=30$ and $\delta =30$, $\Delta t=45$ and $\delta =10$, $\Delta t=45$ and $\delta =30$ respectively. From Fig. 1, it can be seen that the level of the network synchronization fluctuates slightly around 0.36 under different choices of the window size and the time interval between adjacent windows. And the change of both variables does not cause significant change of the network synchronization. In fact, the mean and the standard deviation in Fig. 1a–d are 0.3657 and 0.0478, 0.3658 and 0.0462, 0.3635 and 0.0446, 0.3634 and 0.0446 respectively. Figure 1 also reflects that the financial market exhibit time-varying integration, which is consistent with the results of Bekaert and Harvey (1995) and those of Liu and Tse (2012).

### Regression analysis results

**Table 1:** Descriptive statistics and ADF test

	*S*	*C*	*l*	*μ*	$\Delta \sigma$
Mean	0.3657	0.0234	0.3621	0.0012	0.00003
Max.	0.4961	0.0422	0.4957	0.0127	0.0128
Min.	0.2790	0.0133	0.2784	−0.0117	−0.0086
SD	0.0478	0.0062	0.0461	0.0051	0.0041
ADF-statistic	−2.5941 (0.0971)	−2.9531 (0.0426)	−2.7512 (0.0687)	−2.4036 (0.0163)	−6.6429 (0.0000)

The numbers in parentheses refer to p values

**Table 2:** Correlation coefficients between variables and VIF coefficients

	*C*	*l*	$\mu$	$\Delta \sigma$	*VIF*
*C*	1				73.7770
*l*	0.9931	1			73.7230
$\mu$	−0.5121	−0.4921	1		1.004
$\Delta \sigma$	0.1416	0.1369	−0.1550	1	1.003

**Table 3:** Causality test results

Null hypothesis	F-statistic	Prob.
*S* does not granger cause *C*	2.70641	0.0341
*C* does not granger cause *S*	2.25770	0.0677
*S* does not granger cause *l*	2.71898	0.0335
*l* does not granger cause *S*	2.77096	0.0309

Before the regression analysis, we conduct some tests on the variables. We only provide the test results in the case of $\Delta t=30$ and $\delta =10$. As for the other three cases, we can conduct similar analysis. First, we apply Augmented Dickey–Fuller test (ADF) to analyze whether there are unit roots for all of the five variables, namely, $S, C, l, \mu$ and $\Delta \sigma$. Table 1 illustrates the test results in the case of $\Delta t=30$ and $\delta =10$. Obviously, all of the variables are stationary. Table 2 reports both the correlation coefficients between the variables and VIF (Variance Inflation Factor) coefficients, where VIF can detect the degree of multicollinearity. From Table 2, it can be seen that *C* is highly correlated with *l*, and VIF values of *C* and *l* are bigger than 10. Therefore, there is a multicollinearity problem in the above regression model. According to the above analysis, we adopt the robust principal components regression method to estimate the above regression model, where this method can not only deal with the multicollinearity problem, but also allows treating data with outliers (Hubert and Verboven 2003). The result of the regression in the case of $\Delta t=30$ and $\delta =10$ is shown in Eq. (8). The estimation results of other three cases are given in Eqs. (9)–(11) respectively.

8 $$S\,=\, 0.0007+0.1222C+0.9969l-0.0238\mu +0.0251\Delta \sigma +\epsilon$$

9 $$S\,=\, 0.0052+0.1224C+0.9843l-0.0598\mu +0.0434\Delta \sigma +\epsilon$$

10 $$S\,=\, 0.0028+0.1184C+0.9861l-0.0178\mu +0.0027\Delta \sigma +\epsilon$$

11 $$S\,=\, 0.0041+0.1260C+0.9822l-0.0567\mu +0.0490\Delta \sigma +\epsilon$$

According to the estimation results of the regression model, we can see that the values of $\alpha _{1}$ and $\alpha _{2}$ are positive, which means the clustering coefficient and the average shortest path length have a positive effect on the collective behavior respectively. This happens because the bigger the clustering coefficient and the average shortest path length are, the higher correlation coefficients among stock price series are. Therefore, stocks with similar time series and stock prices tend to exhibit similar evolution. Besides, we can know that more similar time evolution of stock prices imply higher the clustering coefficient and the average shortest path length of the stock market network based on Eqs. (5) and (6). This means there exists bidirectional relationships between the collective behavior and network properties. In fact, this can be confirmed through Granger causality tests. Granger (1969) defines causality between two variables in terms of predictability. In Table 3, we report the results of Granger causality tests. From it we can see that there are bidirectional relationships between the collective behavior and network properties. Peron and Rodrigues (2011) find that the clustering coefficient and the average shortest path length have a negative effect on the collective behavior by using the data from New York Stock Exchange. Comparing with the results of Peron and Rodrigues (2011), we can find that our results of the effect of the clustering coefficient and the average shortest path length on the collective behavior are different. The possible reasons are that the data analyzed are different, that they only analyze the effect of network structures, while we take dynamic properties of the financial market into consideration.

From Eqs. (8)–(11), it can be seen that the value of $\alpha _{3}$ is negative and that of $\alpha _{4}$ is positive. This means that the average return has a negative effect on the collective behavior, while the volatility fluctuation has a positive effect on it. As for the effect of stock market properties on network synchronization, Liu and Tse (2012) adopt the Pearson’s correlation, and find that network synchronization is positively correlated with volatility, and is negatively correlated with average stock prices. Compared with the study of Liu and Tse (2012), in this paper stock market properties analyzed are average return and volatility fluctuation. Besides, network nodes in the study of Liu and Tse (2012) are stock markets, while network nodes in this paper are stocks.

From Eqs. (8)–(11), we know that the value of $\alpha _{2}$ is larger than the values of $\alpha _{1}$, $\alpha _{3}$ and $\alpha _{4}$. This means that the major influence on the collective behavior is due to the average shortest path length. In addition, the change of both the window size and the time interval between adjacent windows does not cause significant changes in the estimation results of the regression model. Therefore, the above results are robust against the window size and the time interval between adjacent windows.

12 $$S\,=\,-0.0033+0.1195C+1.0113l-0.0117\mu +0.1146\Delta \sigma +\epsilon$$

13 $$S\,=\, -0.0054+0.1393C+1.0151l-0.1046\mu +0.0034\Delta \sigma +\epsilon$$

14 $$S\,=\, -0.0008+0.1233C+0.9998l-0.0177\mu +0.0697\Delta \sigma +\epsilon$$

Now we analyze the effect of the latest financial crisis 2007–2008 on the above results. In the case of $\Delta t=30$ and $\delta =10$, we estimate Eq. (4) by using the data in three periods respectively, where the three periods are the period of the normal stock market, the period of the financial crisis and the period of the stock market recovery. And their estimation results are shown in Eqs. (12)–(14) respectively. From Eqs. (12)–(14), it can be seen that the effect of network structures and stock market properties on the collective behavior during the financial crisis is the same as those during other periods.

## Conclusion

Financial markets are complex systems, and can be represented as complex networks. In this paper, we construct financial market networks based on the data from the Chinese financial market, and then analyze the determination of the collective behavior based on the network synchronization. We adopt a regression model to determine how factors, the structural properties and financial market properties, influence the collective behavior.

First, the regression results show that the structural properties, the clustering coefficient and the average shortest path length, have a positive effect on the collective behavior. With respect to financial market properties, we find that the average return has a negative effect on the collective behavior, while the volatility fluctuation has a positive effect on the collective behavior. Second, the effect of the average shortest path length on the collective behavior is the greatest in the four variables. Third, we find that the above results are robust against the window size and the time interval between adjacent windows. Beside, the effect of network structures and stock market properties on the collective behavior during the financial crisis is the same as those during other periods.

In summary, the results in this paper would be useful in understanding the emergence of collective behavior in the financial marker from the network perspective. The above results imply that stock price correlations should be considered in the process of investment portfolio decision and risk management, and network synchronization can be as a useful tool in measuring the collective behavior of the financial market. And we should take topological features into consideration besides stock returns and volatility.
